# Supplementary material for: Accelerating Bayesian inference of dependency between mixed-type biological traits
Source: PLoS Comput Biol. 2023 Aug 28;19(8):e1011419. doi: 10.1371/journal.pcbi.1011419 (PMC10491301; doi:10.1371/journal.pcbi.1011419)
Supplement: S1 File — A heuristic to auto-tune the step size ratio r. (PDF) [file pcbi.1011419.s001.pdf]

Auto-tuning of r

We describe a simple heuristic to auto-tune the step size ratio r on the fly. Let $\mathbf{\Sigma}_G$ and $\mathbf{\Sigma}_L$ be the covariance matrices for $\boldsymbol{x}_G$ and $\boldsymbol{x}_L$ respectively, then their minimal eigenvalues $\lambda_{\min,G}$ and $\lambda_{\min,L}$ describe the variance magnitude in the most constrained direction. Intuitively, for both HMC and Zigzag-HMC, the step size should be proportional to the diameter of this most constrained density region, which is $\sqrt{\lambda_{\min,G}}$ or $\sqrt{\lambda_{\min,L}}$. Therefore we propose a choice of $r = \sqrt{\lambda_{\min,L}}/\sqrt{\lambda_{\min,G}}$, assuming the two types of momenta lead to similar travel distance during one unit time. It is straightforward to check this assumption. At stationarity, HMC has a velocity $\boldsymbol{v}_G \sim \mathcal{N}(\mathbf{0}, \mathbf{I})$, so its velocity along any unit vector $\boldsymbol{u}$ would be distributed as $\langle \boldsymbol{v}_G, \boldsymbol{u} \rangle \sim \mathcal{N}(0, 1)$, and the travel distance $\mathbb{E}|\langle \boldsymbol{v}_G, \mathbf{u} \rangle| = \sqrt{2/\pi}$. For Zigzag-HMC, as $\langle \boldsymbol{v}_L, \boldsymbol{u} \rangle$ does not follow a simple distribution, we estimate $\mathbb{E}|\langle \boldsymbol{v}_L, \mathbf{u} \rangle|$ by Monte Carlo simulation and it turns out to be ≈ 0.8, close to $\sqrt{2/\pi}$.

We test this intuitive choice of r on a subset of the HIV data in [1] with 535 taxa, 5 binary and 3 continuous traits. We calculate the optimal $r = \sqrt{\lambda_{\min,L}}/\sqrt{\lambda_{\min,G}} \approx 2.5$ with $\mathbf{\Sigma}_G$ and $\mathbf{\Sigma}_L$ estimated from the MCMC samples. Clearly, r has a significant impact on the efficiency as a very small or large r leads to lower ESS (Table 1). Also, an r in the order of our optimal value generates the best result, so we recommend this on-the-fly automatic tuning $r = \sqrt{\lambda_{\min,L}}/\sqrt{\lambda_{\min,G}}$ (Table 1).

Table 1: Minimal effective sample size (ESS) per running hour (hr) for partial correlation matrix elements r_{ij} with different r ($N = 535, P_{\text{disc}} = 5, P_{\text{cont}} = 3$). ESS values report medians across 3 independent simulations.

	ESS/hr	
r	min	median
0.1	32	266
1	106	771
10	**118**	**855**
100	25	110

References

1. Zhang Z, Nishimura A, Bastide P, Ji X, Payne RP, Goulder P, et al. Large-scale inference of correlation among mixed-type biological traits with phylogenetic multivariate probit models. The Annals of Applied Statistics. 2021;15(1):230–251.
