# Supplementary material for: Accelerating Bayesian inference of dependency between mixed-type biological traits
Source: PLoS Comput Biol. 2023 Aug 28;19(8):e1011419. doi: 10.1371/journal.pcbi.1011419 (PMC10491301; doi:10.1371/journal.pcbi.1011419)
Supplement: S2 File — An intuition for BPS’s slow movement in energy space. (PDF) [file pcbi.1011419.s002.pdf]

Zigzag-HMC explores the energy space more efficiently than BPS

In our experience, BPS tends to generate samples with high auto-correlation between their respective energy function evaluations $-\log \pi(\boldsymbol{x})$. In other words, it slowly traverses the target distribution's energy contours even when the marginal dimensions all appear to demonstrate good mixing. A similar behavior has also been reported by [2], who introduce a velocity refreshment to address the issue. As we demonstrate below, however, even velocity refreshments cannot fully remedy BPS's slow-mixing on the energy space.

We apply BPS and Zigzag-HMC to a 256-dimensional standard normal truncated to the positive orthant (all $x_i > 0$). We run both samplers for 2000 iterations where per-iteration travel time is one unit time interval and repeat the experiments for 10 times with varying initial values. For BPS we include Poisson velocity refreshments to avoid reducible behavior and set the refreshment rate to an optimal value 1.4 [3]. At every iteration we refresh Zigzag-HMC's momentum by redrawing it from the marginal Laplace distribution. Both samplers have no problem sampling from the target distribution and the minimal ESS across all dimensions are 158 ± 25 (mean $\pm$ SD) for BPS and 207 ± 21 for Zigzag-HMC, estimated from the last 1000 samples of the MCMC chains across 10 runs. As a sanity check, the average sample mean and variance are $(0.800, 0.365)$ for BPS and $(0.798, 0.363)$ for Zigzag-HMC, close to the analytical values — the univariate marginal distribution of our truncated standard normal is a truncated normal with mean $2/\sqrt{2\pi} \approx 0.798$ and variance $1 - 2/\pi \approx 0.363$ [4].

However, Zigzag-HMC returns a clear win over BPS in the mixing of joint density (Figure 1). The sampling inefficiency for $-\log \pi(\boldsymbol{x})$ is less of a problem if one only needs to sample from a truncated normal with a fixed covariance matrix, but we are keenly interested in sampling the covariance matrix as a target of scientific interest. In this context, inefficient traversal across energy contours harms the sampling efficiency for all model parameters.

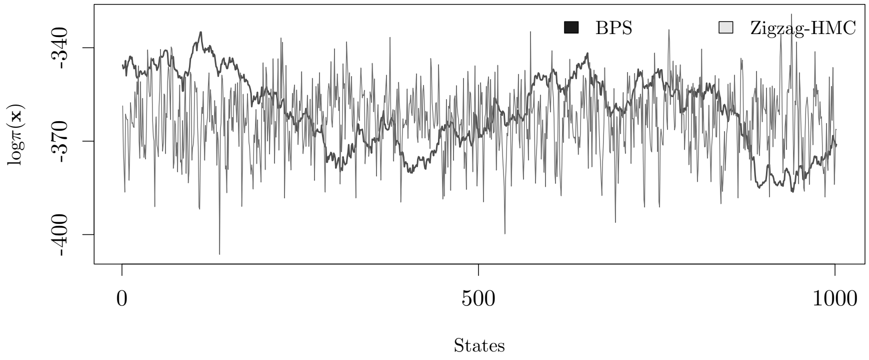

Figure 1: Trace plot of the log density of a 256-dimensional truncated standard normal sampled by BPS and Zigzag-HMC for 1000 MCMC iterations.

We can provide an intuition for BPS's slow movement in energy space. Assume the d-dimensional parameter at the tth MCMC iteration is $\boldsymbol{x}(t) = (x_1(t), \ldots, x_d(t)) \in \mathbb{R}^d$, $t = 1, \ldots, T$, with T being the total number of iterations. For a truncated standard normal, its log density $\log \pi(\boldsymbol{x}) \propto \sum_i^d x_i^2$, and a high auto-correlation suggests $\log \pi(\boldsymbol{x})$ changes little between successive iterations, that is, the squared jumping distances

$$J_D = \left[\sum_i^d x_i^2(t+1) - \sum_i^d x_i^2(t) \right]^2, \quad t = 0, \ldots, T-1$$

are small. We then decompose J_D into two components

$$
\begin{aligned}
J_D &= J_1 + J_2, \\
J_1 &= \sum_i^d \left[x_i^2(t+1) - x_i^2(t)\right]^2, \\
J_2 &= \sum_{j \neq k}^d \left[x_j^2(t+1) - x_j^2(t)\right] \left[x_k^2(t+1) - x_k^2(t)\right], \; t = 0, \ldots, T-1,
\end{aligned}
\tag{1}
$$

where J_1 measures the sum of the marginal travel distances and J_2 the covariance among them. We compare J_D, J_1 and J_2 between BPS and Zigzag-HMC in the aforementioned experiments. Clearly seen in Table 1, BPS yields a much lower J_D than Zigzag-HMC because its J_2 is largely negative, suggesting strong negative correlation among the coordinates.

Table 1: Squared jumping distance (J_D) of $\log \pi(\boldsymbol{x})$ sampled by the bouncy particle sampler (BPS) and Zigzag Hamiltonian Monte Carlo (Zigzag-HMC). We report the empirical mean of J_1 and J_2 in their means and standard deviations (SD) across ten independent simulations with $T = 1000$ after burn-in samples. Both samplers have a per-iteration travel time 1.

	BPS		Zigzag-HMC	
Quantity	mean	SD	mean	SD
J_D	8.3	0.6	583	21.7
J_1	521	16.3	564	2.6
J_2	-513	16.0	18.6	20.9

References

1. Zhang Z, Nishimura A, Bastide P, Ji X, Payne RP, Goulder P, et al. Large-scale inference of correlation among mixed-type biological traits with phylogenetic multivariate probit models. The Annals of Applied Statistics. 2021;15(1):230–251.

2. Bouchard-Côté A, Vollmer SJ, Doucet A. The bouncy particle sampler: A nonreversible rejection-free Markov chain Monte Carlo method. Journal of the American Statistical Association. 2018;113(522):855–867.

3. Bierkens J, Kamatani K, Roberts GO. High-dimensional scaling limits of piecewise deterministic sampling algorithms. arXiv preprint arXiv:180711358. 2018;.

4. Cartinhour J. One-dimensional marginal density functions of a truncated multivariate normal density function. Communications in Statistics-Theory and Methods. 1990;19(1):197–203.
