# Supplementary material for: Accelerating Bayesian inference of dependency between mixed-type biological traits
Source: PLoS Comput Biol. 2023 Aug 28;19(8):e1011419. doi: 10.1371/journal.pcbi.1011419 (PMC10491301; doi:10.1371/journal.pcbi.1011419)
Supplement: S1 Fig — (PDF) [file pcbi.1011419.s003.pdf]

Fig S1. Histograms of per run-time ESS for r_{ij}

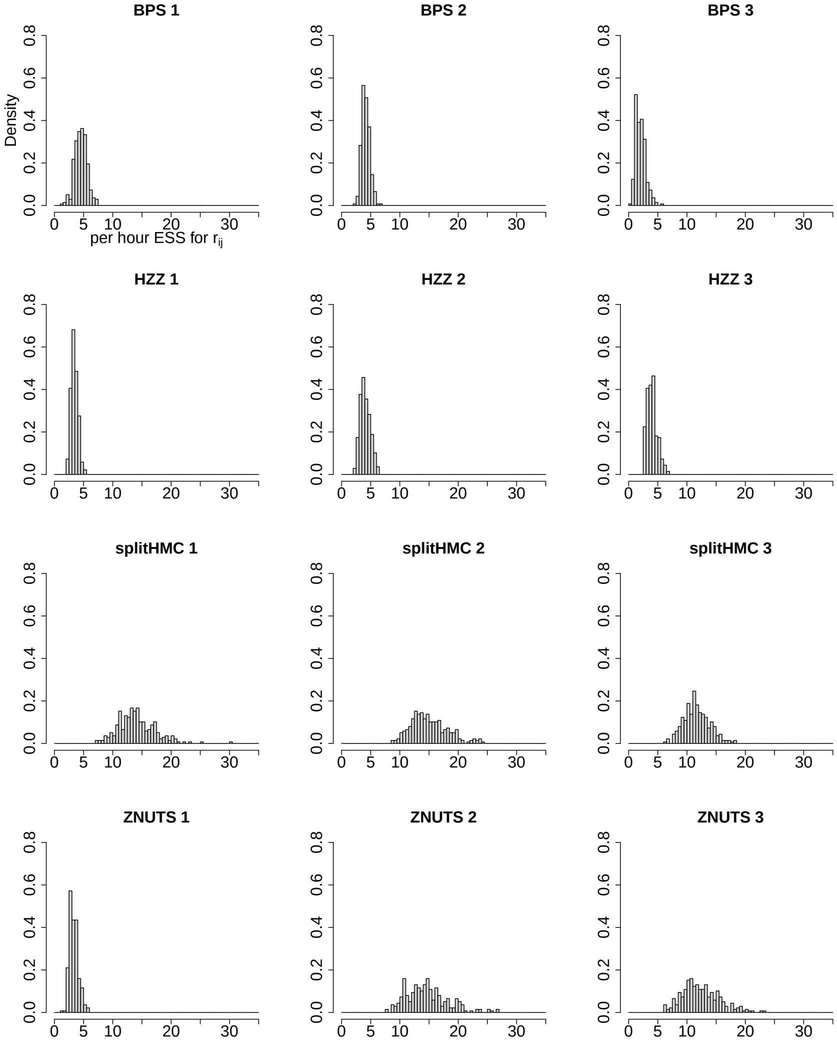

Fig S1: Histograms of per-hour run-time ESS for **R** elements, as discussed in Section "Efficiency gain from the new inference scheme". Each sampling scheme includes three independent repeats (1, 2, 3), and the x-axis and y-axis are consistent across all subfigures.
